# Mapping the mortality niche of chronic lower respiratory diseases complicated by respiratory failure: a multiscale spatial analysis of environmental and social drivers

**DOI:** 10.3389/fpubh.2026.1819428

**Published:** 2026-05-26

**Authors:** Dong Miao, Jingyan Li, Xinfeng Bai, Zhiyong Song, Jun Gan, XiaoBing Hu, Wei Chen, Yingping Tian, Xiaolei Ye, Junfeng Zheng

**Affiliations:** 1 Department of Emergency, The 980th Hospital of the PLA Joint Logistics Support Force (Primary Bethune International Peace Hospital of PLA), Shijiazhuang, China; 2 Department of Emergency, The Second Hospital of Hebei Medical University, Shijiazhuang, China; 3 Department of Intensive Care Medicine, Hebei Children's Hospital of Hebei Medical University, Shijiazhuang, China; 4 The Center for Disease Prevention and Control in Western Theater Command of PLA Joint Logistic Support Force, Lanzhou, China; 5 Department of Emergency, The Third Medical Center of Chinese PLA General Hospital, Beijing, China

**Keywords:** chronic lower respiratory diseases, ecological niche, multiscale geographically weighted regression, respiratory failure, social vulnerability, spatial heterogeneity

## Abstract

**Background:**

Chronic lower respiratory diseases complicated by respiratory failure as a multiple cause of death, denoted as RF-CLRD, represent a critical public health challenge with significant spatial heterogeneity across the United States. Although bioclimatic factors, air pollution, and social vulnerability are known determinants of respiratory health, traditional global models assume these risk factors operate uniformly across space, obscuring their spatial non-stationarity and region-specific impacts.

**Methods:**

We utilized county-level data from the contiguous United States (2010–2019) to quantify mortality rates of RF-CLRD complicated by respiratory failure. Spatial clustering was evaluated using Moran's I and Local Indicators of Spatial Association (LISA) statistics. To capture process spatial heterogeneity, we developed a Multiscale Geographically Weighted Regression (MGWR) model integrating bioclimatic, pollution, and socioeconomic variables.

**Results:**

RF-CLRD mortality rates exhibited marked spatial heterogeneity with multi-centric clustering across the contiguous United States. High-mortality hotspots were not limited to the Appalachian and Ozark highlands and the Deep South, but also formed distinct clusters in the Intermountain West and Northern Maine. The MGWR model significantly outperformed global OLS (Adjusted *R*^2^: 0.66 vs. 0.39), effectively capturing non-stationarity. The analysis identified a dual-scale mechanism: bioclimatic factors, PM_2.5_, and social vulnerability acted as broad, stable stressors (global scale), whereas population aging and smoking prevalence operated as localized amplifiers (regional scale). Notably, MGWR revealed that smoking's impact was spatially variable—strong in northern and coastal peripheries but insignificant in the South Central region.

**Conclusion:**

The spatial heterogeneity of RF-CLRD mortality rates arises from the interplay between a stable macro-environmental niche and localized demographic amplifiers. This scale-dependent structure necessitates a transition from uniform national strategies to place-based precision policies. Interventions should prioritize climate adaptation and pollution mitigation in environmentally vulnerable regions where structural risks dominate, while targeting smoking cessation and geriatric care in specific behavioral hotspots.

## Introduction

Chronic lower respiratory diseases (CLRD) represent a formidable public health challenge, currently ranking as the third leading cause of death globally (1). While the underlying pathology of CLRD is progressive, the terminal trajectory for many patients is often precipitated by acute exacerbations that evolve into respiratory failure, a critical condition characterized by inadequate gas exchange (2). In the United States, despite advances in clinical management, the mortality burden of CLRD complicated by respiratory failure exhibits significant spatial heterogeneity (3). These geographical disparities suggest that beyond individual risk factors, the external environment—specifically the “climatic niche” in which populations reside—plays a pivotal determinant role in survival outcomes.

Although the influence of meteorological factors on respiratory health is well-documented, traditional epidemiology has predominantly focused on single-variable extremes such as heatwaves or cold spells, overlooking the complex multivariate environment of human exposure (4). The concept of a “climatic niche,” adapted from ecological modeling, offers a novel framework to understand environmental suitability for disease transmission (5). For patients with compromised lung function, specific combinations of bioclimatic factors (e.g., low temperature combined with low humidity) may synergistically impair mucociliary clearance and airway defense mechanisms, thereby lowering the threshold for fatal respiratory failure (6). Crucially, this climatic influence interacts with anthropogenic pollution and social determinants, where fine particulate matter acts as a systemic inflammatory agent amplifying meteorological stressors and social vulnerability acts as a critical effect modifier under the “triple jeopardy” hypothesis (7–9).

Consequently, the risk of dying from respiratory failure is a product of the interaction between bioclimatic exposure, air quality, and socioeconomic resilience, yet traditional global regression models often mask these local variations by assuming spatial stationarity. To address this limitation, this study employs MGWR to capture spatial non-stationarity (10), aiming to map the climatic mortality niche of RF-CLRD across the contiguous United States from 2010 to 2019 and identify region-specific drivers to inform targeted public health resource allocation.

## 2 Materials and methods

### 2.1 Data sources and processing

#### 2.1.1 Mortality and population data

We extracted county-level mortality records for the 48 contiguous United States from 2010 to 2019 using the Centers for Disease Control and Prevention (CDC) WONDER Multiple Cause of Death database. This specific decadal period was deliberately selected to establish a clean, long-term epidemiological baseline prior to the unmanageable systemic confounding effects introduced by the COVID-19 pandemic. The outcome of interest was defined as death where the underlying cause was Chronic Lower Respiratory Disease (ICD-10 codes J40-J47) and the multiple cause included Respiratory Failure (ICD-10 code J96). Crude mortality rates (CMR) were calculated based on the aggregated death counts and population estimates over the 10-year period to stabilize stochastic variance and mitigate the “small numbers problem” inherent in county-level spatial epidemiology (11, 12). To ensure data reliability, counties with suppressed records (fewer than 10 deaths) were excluded, resulting in a baseline sample of 2,731 counties for descriptive analysis. For regression modeling, the CMR was natural log-transformed to normalize the distribution; consequently, 20 counties with zero reported deaths were removed, yielding a final analytical sample of 2,711 counties. To account for the use of crude rates, the percentage of the population aged 65+ (derived from the SVI demographic dataset) was included as an obligatory covariate to adjust for age structure.

#### 2.1.2 Environmental exposures

To characterize the “climatic niche,” we generated 19 standard bioclimatic variables (BioClim) representing annual trends, seasonality, and extreme environmental conditions (detailed definitions are provided in Supplementary Table S1). Since pre-calculated BioClim data were unavailable for the specific 2010–2019 study period, we acquired raw monthly meteorological rasters (minimum temperature, maximum temperature, and precipitation) at 4-km resolution from the PRISM Climate Group (Oregon State University). These monthly data were processed pixel-by-pixel to compute the 19 BioClim indicators, capturing the precise climatological exposure during the study decade. Fine particulate matter (PM_2.5_) exposure was derived from the Washington University in St. Louis (WUSTL) V5.GL.02 dataset, which provides high-resolution (0.01° × 0.01°) global estimates. We processed the annual rasters in ArcGIS 10.8 using Cell Statistics to calculate a 10-year mean composite, which effectively captures the persistent structural macro-environmental baseline (the “climatic niche”) rather than transient meteorological noise (13, 14), followed by Zonal Statistics to aggregate the pixel-level data into county-level mean concentrations.

#### 2.1.3 Social and behavioral determinants

Socioeconomic vulnerability was assessed using the Social Vulnerability Index (SVI) from the CDC Agency for Toxic Substances and Disease Registry (ATSDR). Behavioral risk factors, including adult smoking, adult obesity (BMI ≥ 30), and excessive drinking, were obtained from the County Health Rankings & Roadmaps program. Given the periodic and multi-year reporting nature of these datasets, we computed their long-term averages across the available reporting cycles. This approach yields a highly stable socio-behavioral baseline that corresponds to the decadal mortality period while minimizing the stochastic variance of intermittent survey years. These datasets were merged with the mortality and environmental data by unique Federal Information Processing Standards (FIPS) codes to form the final dataset for spatial analysis.

### 2.2 Variable selection and global regression

To identify the primary drivers contributing to RF-CLRD mortality, we initially constructed a comprehensive set of independent variables. This collection included variables representing air pollution exposure (PM_2.5_ concentration), bioclimatic factors (19 bioclimatic variables), socioeconomic and behavioral factors (SVI, smoking prevalence, excessive drinking, and obesity rates), and demographic structure (the percentage of population aged ≥65).

Given the potential multicollinearity among these predictors—particularly between bioclimatic factors and between social/demographic variables—we employed a rigorous stepwise selection process. First, Spearman's rank correlation analysis was performed to examine both the correlations among independent variables and their respective bivariate associations with the dependent variable (RF-CLRD mortality). When any pair of independent variables exhibited a Spearman correlation coefficient exceeding 0.75, the variable demonstrating a weaker bivariate correlation with the dependent variable was systematically excluded, while the stronger predictor was retained. Subsequently, we calculated the Variance Inflation Factor (VIF) for the remaining candidate predictors. Any variable with a VIF greater than 5 was further excluded in a stepwise manner to ensure the stability of the coefficient estimates (15). The final OLS model served as the global baseline, estimating the average linear relationships between the selected environmental, social, and demographic stressors and mortality rates across the contiguous United States.

### 2.3 Spatial autocorrelation and cluster analysis

To validate the underlying assumption of spatial stationarity in the global OLS model and investigate the spatial pattern of mortality, we conducted a two-step spatial autocorrelation analysis. First, the Global Moran's I statistic was calculated to test for overall spatial dependence. A statistically significant positive Moran's I (*p* < 0.05) indicates that mortality rates are not randomly distributed but spatially clustered, thereby violating the Gauss-Markov independence assumption (16). Second, to identify specific local spatial clusters (hotspots and coldspots) and spatial outliers, we employed the univariate LISA statistic (Local Moran's I). This analysis decomposed the global statistic into local contributions, categorizing counties into four spatial regimes (High-High, Low-Low, Low-High, and High-Low) based on a significance level of *p* < 0.05 (17). The resulting LISA cluster map visualized the local spatial heterogeneity of the disease burden.

### 2.4 Multiscale geographically weighted regression

To address the spatial non-stationarity identified in the diagnosis, we advanced from the global OLS model to Geographically Weighted Regression (GWR) and finally to MGWR (18). Unlike traditional GWR, which enforces a single optimal bandwidth for all covariates, MGWR allows for covariate-specific bandwidths. This is critical for our study, as the scale of influence for bioclimatic factors likely differs from that of social vulnerability. We calibrated the MGWR model using an adaptive bisquare spatial kernel, which accommodates the irregular density of US counties. The optimal bandwidths were determined using the Golden Section Search method. Model performance was compared across OLS, GWR, and MGWR using the Corrected Akaike Information Criterion (AICc), adjusted *R*^2^, and residual sum of squares (RSS), with a lower AICc indicating a superior fit to the mortality data (19).

### 2.5 Statistical analysis

All statistical tests were two-sided, and a *p*-value < 0.05 was considered statistically significant. Descriptive statistics for continuous variables are expressed as median with interquartile range (IQR), while categorical variables are presented as frequencies and percentages (%). Spatial data processing and mapping were conducted using ArcGIS 10.8 (ESRI Inc., Redlands, CA, USA), and spatial regression modeling was performed using MGWR 2.2 software.

## 3 Results

### 3.1 Descriptive statistics and baseline characteristics

A total of 2,731 counties were included in the final analysis, covering 99.3% of the contiguous United States population. Table 1 summarizes the descriptive statistics for mortality, environmental exposures, and socioeconomic covariates.

**Table 1:** Baseline characteristics of RF-CLRD mortality, bioclimatic factors, and social vulnerability across the contiguous US (2010–2019).

Variable	Median (IQR)	Range (min–max)
Sample representativeness		
Population representation (%)	99.31 (99.30, 99.32)	99.28, 99.33
Health outcome		
RF-associated CLRD crude mortality rate (per 100 k)	16.99 (11.83, 23.18)	0.00, 98.53
Demographics		
Population aged 65+ (%)	14.01 (12.07, 16.01)	5.79, 43.13
Air pollution exposures		
PM_2.5_ concentration (μg/m3)	7.56 (6.51, 8.24)	3.54, 12.28
Bioclimatic factors		
Annual mean temp (Bio1, °C)	13.03 (9.80, 16.41)	1.39, 24.90
Mean diurnal range (Bio2, °C)	11.85 (11.09, 12.69)	7.14, 18.64
Isothermality (Bio3, Index)	32.94 (28.73, 36.47)	21.83, 54.19
Temp. seasonality (Bio4, SD × 100)	910.64 (812.83, 1,017.02)	215.84, 1,324.34
Max temp warmest month (Bio5, °C)	31.20 (29.04, 33.25)	18.72, 41.45
Min temp coldest month (Bio6, °C)	−6.06 (−10.01, −1.59)	−22.11, 13.33
Temp. annual range (Bio7, °C)	37.08 (34.50, 39.69)	14.22, 48.56
Mean temp wettest qtr (Bio8, °C)	18.07 (15.87, 20.31)	−2.59, 28.38
Mean temp driest qtr (Bio9, °C)	12.53 (4.72, 18.03)	−9.78, 26.38
Mean temp warmest qtr (Bio10, °C)	23.86 (21.54, 26.35)	10.87, 33.03
Mean temp coldest qtr (Bio11, °C)	3.00 (−1.22, 7.70)	−11.47, 20.64
Annual precipitation (Bio12, mm)	1,181.62 (926.73, 1,342.19)	79.56, 2,990.92
Precip. wettest month (Bio13, mm)	204.19 (176.16, 234.43)	29.89, 694.67
Precip. driest month (Bio14, mm)	27.19 (11.58, 35.45)	0.00, 65.38
Precip. seasonality (Bio15, CV)	56.86 (48.49, 74.52)	32.45, 146.39
Precip. wettest qtr (Bio16, mm)	450.92 (392.39, 498.33)	47.14, 1,324.39
Precip. driest qtr (Bio17, mm)	169.28 (88.08, 209.11)	0.24, 353.13
Precip. warmest qtr (Bio18, mm)	329.83 (274.34, 368.72)	0.47, 798.43
Precip. coldest qtr (Bio19, mm)	236.91 (130.68, 303.77)	25.51, 1,237.76
Socioeconomic & behavioral factors		
Social Vulnerability Index (SVI, 0–1)	0.53 (0.29, 0.75)	0.00, 1.00
Current smoking (%)	19.62 (16.95, 22.19)	6.27, 39.53
Excessive drinking (%)	16.81 (14.17, 19.09)	6.86, 30.10
Obesity (%)	32.64 (30.09, 34.79)	14.97, 45.84

The study sample (*N* = 2,731) accounts for 99.3% of the total population of the contiguous United States (3,108 counties). Excluded counties were exclusively those with suppressed mortality data due to small sample sizes.

RF-CLRD, defined as deaths where Chronic Lower Respiratory Disease (ICD-10: J40-J47) was the underlying cause and Respiratory Failure (ICD-10: J96) was listed as a multiple cause of death; IQR, interquartile range; SVI, social vulnerability index; SD, standard deviation; CV, coefficient of variation.

The CMR for RF-CLRD exhibited marked geographic disparity across the study area. The median CMR was 16.99 per 100 k population (IQR: 11.83, 23.18). Notably, the distribution was right-skewed, with county-level mortality rates ranging from 0.00 to a maximum of 98.53 per 100 k, indicating substantial spatial heterogeneity in disease burden. Given this highly skewed distribution (visually confirmed in Supplementary Figure S1), a natural logarithmic transformation was applied to normalize the data for subsequent regression analyses.

Bioclimatic and pollution variables reflected the diverse environmental gradients of the continental US. The annual mean temperature (Bio1) had a median of 13.03 °C (IQR: 9.80, 16.41), while annual precipitation (Bio12) showed a median of 1,181.62 mm (IQR: 926.73, 1,342.19). Regarding air quality, the median annual PM_2.5_ concentration was 7.56 μg/m^3^ (IQR: 6.51, 8.24), with peak exposure levels reaching 12.28 μg/m^3^ in the most polluted counties.

The study population was characterized by significant socioeconomic and behavioral variability. The median SVI was 0.53 (IQR: 0.29, 0.75). Demographic analysis revealed an aging population structure, with a median of 14.01% (IQR: 12.07, 16.01) of residents aged 65+. Behavioral risk factors were prevalent, with median prevalences of 32.64% (IQR: 30.09, 34.79) for obesity, 19.62% (IQR: 16.95, 22.19) for smoking, and 16.81% (IQR: 14.17, 19.09) for excessive drinking.

### 3.2 Spatial patterns of mortality

**Figure 1:**
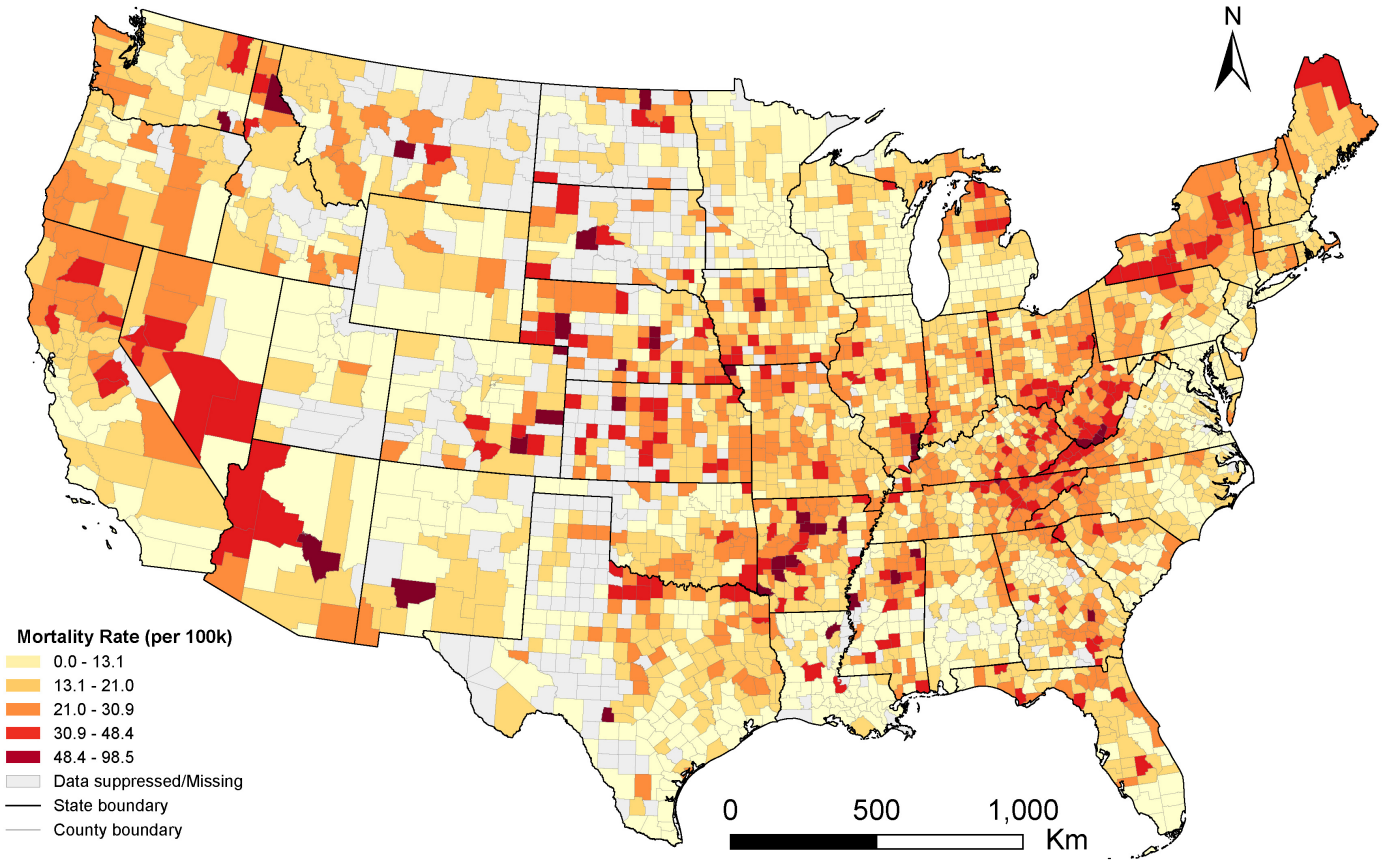
Spatial distribution of average annual crude mortality rates for RF-CLRD across the contiguous United States from 2010 to 2019. This map utilizes CDC WONDER records to illustrate the mortality burden across 2,731 counties based on crude rates per 100 k population while white areas represent regions excluded due to data suppression or missing values.

Figure 1 illustrates the county-level spatial distribution of RF-CLRD mortality across the contiguous United States. Visual inspection reveals a distinct non-stationary pattern characterized by multi-centric spatial clustering rather than a simple gradient. The most prominent high-mortality belt (mortality rates >48.4 per 100 k, indicated in dark red) forms a semi-contiguous corridor extending from Central Appalachia (particularly Kentucky and West Virginia) through the Deep South (Mississippi and Alabama) and into the Ozark and Ouachita Highlands (Arkansas, southern Missouri, and eastern Oklahoma). Beyond this eastern concentration, substantial high-mortality clusters are also evident in the Intermountain West, specifically covering large portions of Nevada and northern Arizona, as well as in Northern Maine. Conversely, lower mortality rates (< 21.0 per 100 k) were generally observed across the Great Plains, the urban Northeast corridor, and parts of the Upper Midwest. This marked spatial heterogeneity in which high-risk clusters coexist with low-risk regions highlights the necessity of employing local spatial modeling techniques including MGWR to better capture region-specific drivers compared to global models.

### 3.3 Spatial clustering analysis

**Figure 2:**
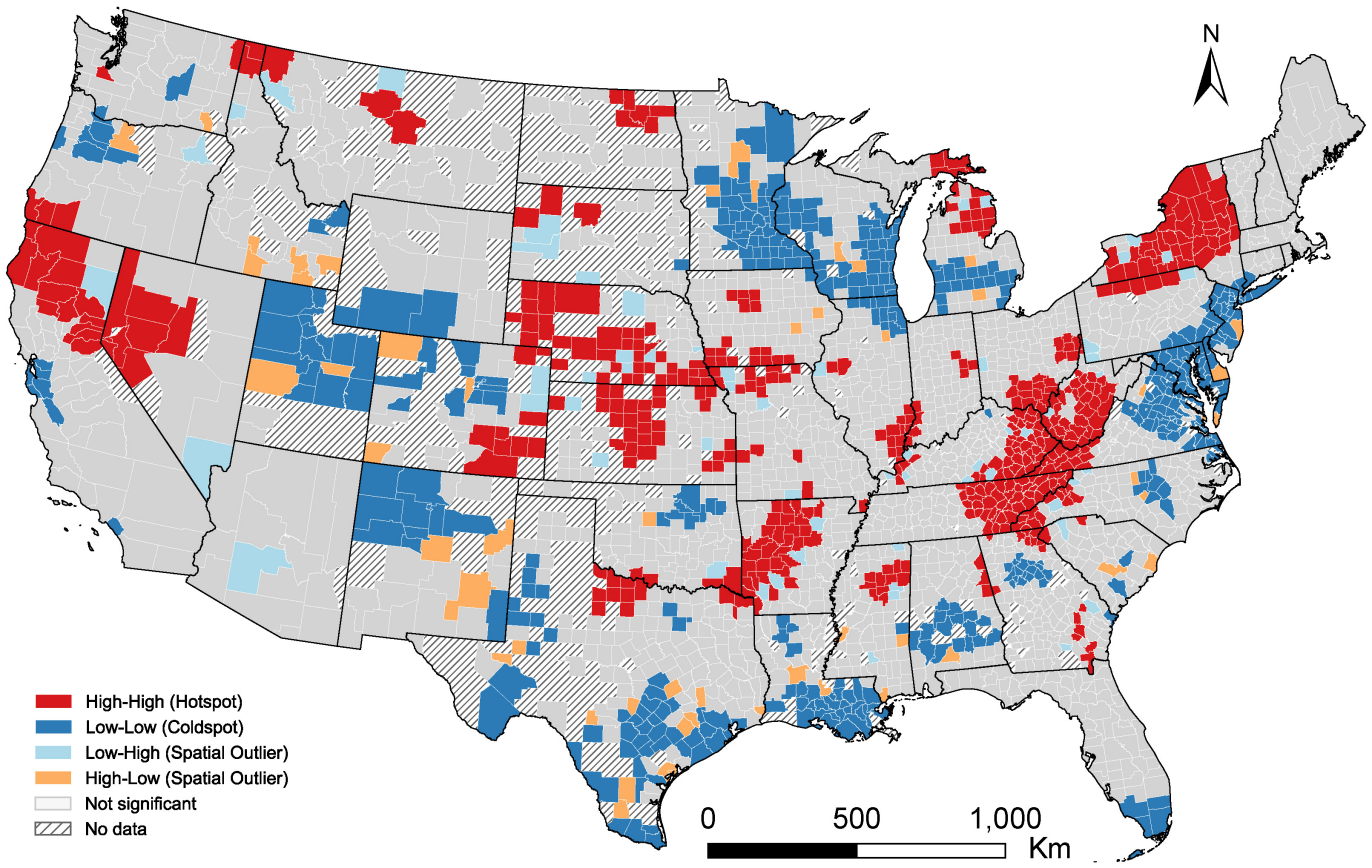
LISA cluster map of log-transformed crude mortality rates for RF-CLRD. The map identifies significant spatial clusters and outliers (*p* < 0.05). Red areas (High-High) indicate hotspots where counties with high mortality rates are surrounded by other high-mortality counties. Dark blue areas (Low-Low) represent coldspots. Light blue (Low-High) and orange (High-Low) areas indicate spatial outliers. Gray areas indicate counties with data but no significant spatial autocorrelation. Counties with zero crude mortality rates, which were excluded from the log-transformed analysis, are shown together with officially suppressed or missing data counties as hatched regions.

To quantify the spatial dependence of RF-CLRD mortality, univariate local Moran's I analysis was performed. The global Moran's I index was 0.469 (*p* < 0.05), confirming significant positive spatial autocorrelation. The LISA cluster map (Figure 2) identifies statistically significant spatial clusters. High-High clusters (hotspots), depicted in red, were predominantly contiguous in the Appalachian region, the Deep South, and parts of the Northeast, aligning with the high-mortality areas observed in Figure 1. Conversely, Low-Low clusters (coldspots), shown in blue, were primarily located in the Great Plains and the Intermountain West. This significant spatial non-stationarity invalidates the independence assumption of traditional regression, necessitating spatial modeling approaches.

### 3.4 Variable selection and model performance

Prior to regression modeling, a rigorous variable selection process was conducted to mitigate multicollinearity. Spearman's rank correlation analysis was first applied to the candidate environmental and socioeconomic predictors (Supplementary Figure S2). Variable pairs exhibiting strong inter-correlations (*|r|* > 0.75) were identified, particularly among bioclimatic temperature and precipitation indices. For these highly collinear pairs, the variables demonstrating weaker bivariate associations with mortality were systematically excluded. The complete details of this stepwise exclusion process, including the specific rationale for dropping each candidate variable, are comprehensively documented in Supplementary Table S2. To further refine the model, VIF analysis was performed on the remaining covariates, the final model retained 13 independent variables. Multicollinearity was effectively minimized, with all VIF values falling well below the threshold of 5, ranging from 1.20 for Aged 65+ to 4.34 for the Mean Temperature of Wettest Quarter (Bio8), thereby ensuring the stability of coefficient estimates in subsequent regression analyses.

Subsequently, the performance of the global OLS model was compared against local spatial models (GWR and MGWR) to identify the optimal modeling strategy (Table 2). The OLS model served as a baseline but showed limited explanatory power (Adjusted *R*^2^ = 0.390) and significant residual spatial autocorrelation (Moran's *I* = 0.343, *p* = 0.001), indicating a failure to capture spatial non-stationarity. In contrast, local models demonstrated superior fit. The GWR model improved the Adjusted *R*^2^ to 0.638. However, MGWR achieved the optimal performance, yielding the highest explanatory power (Adjusted *R*^2^ = 0.660) and the lowest AICc (5226.850), significantly outperforming both OLS and GWR (Δ AICc > 287). Crucially, MGWR effectively eliminated spatial dependence in residuals (Moran's *I* = −0.085), confirming its robustness as the final modeling framework.

**Table 2:** Comparison of model performance and diagnostics between OLS, GWR, and MGWR.

Diagnostic criteria	OLS	GWR	MGWR
Goodness-of-fit			
*R* ^2^	0.393	0.697	0.706
Adjusted *R^2^*	0.390	0.638	0.660
AICc	6,371.677	5,514.294	5,226.850
RSS	1,646.437	822.431	796.808
Model complexity			
ENP (Effective no. of parameters)	15.000	440.245	347.341
σ (Residual Std. Error)	0.782	0.602	0.583
Spatial robustness			
Residual Moran's I	0.343	0.021	−0.085
*P*-value of residual Moran's I	0.001	0.012	0.001

*R*^2^ and Adjusted R^2^ represent the proportion of variance explained by the model, adjusted for the number of predictors and complexity. A lower AICc value indicates a better-fitting model with a penalty for complexity; a difference of >12 is considered statistically significant. Residual Moran's I measures the spatial autocorrelation of model residuals using a K-nearest neighbor (*k* = 8) weight matrix; values closer to 0 indicate a random distribution of residuals, confirming the mitigation of spatial bias. The p-values for Moran's I were obtained via 999 permutations.

OLS, ordinary least squares; GWR, geographically weighted regression; MGWR, multiscale geographically weighted regression; AICc, corrected Akaike information criterion; RSS, residual sum of squares; ENP, effective number of parameters; σ, residual standard error.

### 3.5 Spatial scales and coefficient heterogeneity

Table 3 highlights the multiscale nature of mortality drivers. MGWR identified distinct spatial bandwidths: the intercept operated at a strictly local scale (24 counties), while behavioral and demographic factors like smoking and Age 65+ operated regionally (414–475 counties). In contrast, environmental variables and SVI exhibited global stability (=2,710 counties).

**Table 3:** Comparison of standardized coefficients (β) and spatial scales for OLS and MGWR models.

Variable	OLS (Global)		MGWR (local)		
	Coef. (β)	*P*	Mean β ±SD	Range [min, max]	Bandwidth (scale)
Intercept	0.00	1.00	0.05 ± 0.63	[−1.85, 2.04]	24 (Local)
Air pollution exposures					
PM_2.5_	0.12	< 0.01	0.02 ± 0.00	[0.02, 0.03]	2,710 (Global)
Bioclimatic factors					
Bio2 (Mean Diurnal Range)	0.16	< 0.01	−0.10 ± 0.00	[−0.10, −0.10]	2,710 (Global)
Bio5 (Max Temp Warmest Month)	−0.07	0.02	0.08 ± 0.00	[0.07, 0.09]	2,710 (Global)
Bio7 (Temp. Annual Range)	0.11	< 0.01	0.32 ± 0.00	[0.32, 0.33]	2,710 (Global)
Bio8 (Mean Temp Wettest Qtr)	0.04	0.27	0.08 ± 0.01	[0.07, 0.12]	2,689 (Global)
Bio14 (Precip. Driest Month)	0.03	0.23	−0.02 ± 0.00	[−0.03, −0.02]	2,710 (Global)
Bio19 (Precip. Coldest Qtr)	0.09	0.00	0.09 ± 0.00	[0.07, 0.09]	2,710 (Global)
Bio18 (Precip. Warmest Qtr)	−0.15	< 0.01	0.14 ± 0.01	[0.13, 0.16]	2,710 (Global)
Socioeconomic & behavioral factors					
Social vulnerability index (SVI, 0–1)	−0.02	0.45	0.06 ± 0.01	[0.04, 0.06]	2,601 (Global)
Current smoking (%)	0.31	< 0.01	0.22 ± 0.09	[−0.04, 0.35]	414 (Regional)
Excessive drinking (%)	−0.02	0.50	0.03 ± 0.00	[0.02, 0.03]	2,710 (Global)
Obesity (%)	0.03	0.27	0.08 ± 0.00	[0.08, 0.09]	2,710 (Global)
Demographics (control)					
Population aged 65+ (%)	0.46	< 0.01	0.43 ± 0.09	[0.21, 0.58]	475 (Regional)
Model diagnostics					
AICc	6,373.70	—	5,226.85	—	—
Adjusted *R^2^*	0.39	—	0.66	—	—

All coefficients are standardized (β). Bandwidths indicate the spatial scale of the processes: Local indicates high spatial heterogeneity (small bandwidth), Regional indicates intermediate scales, and Global indicates spatial stationarity.

Crucially, MGWR corrected misleading global estimates found in OLS. While Age 65+ remained the dominant predictor (β = 0.43), the local model revealed coefficient sign reversals for mean diurnal range (Bio2) and max temperature of warmest month (Bio5), uncovering relationships masked by global averaging. Additionally, SVI and Obesity, which were statistically insignificant in OLS (*p* > 0.05), demonstrated positive mean associations in the MGWR framework, validating their local relevance to respiratory mortality.

### 3.6 Spatial variation of key determinants

**Figure 3:**
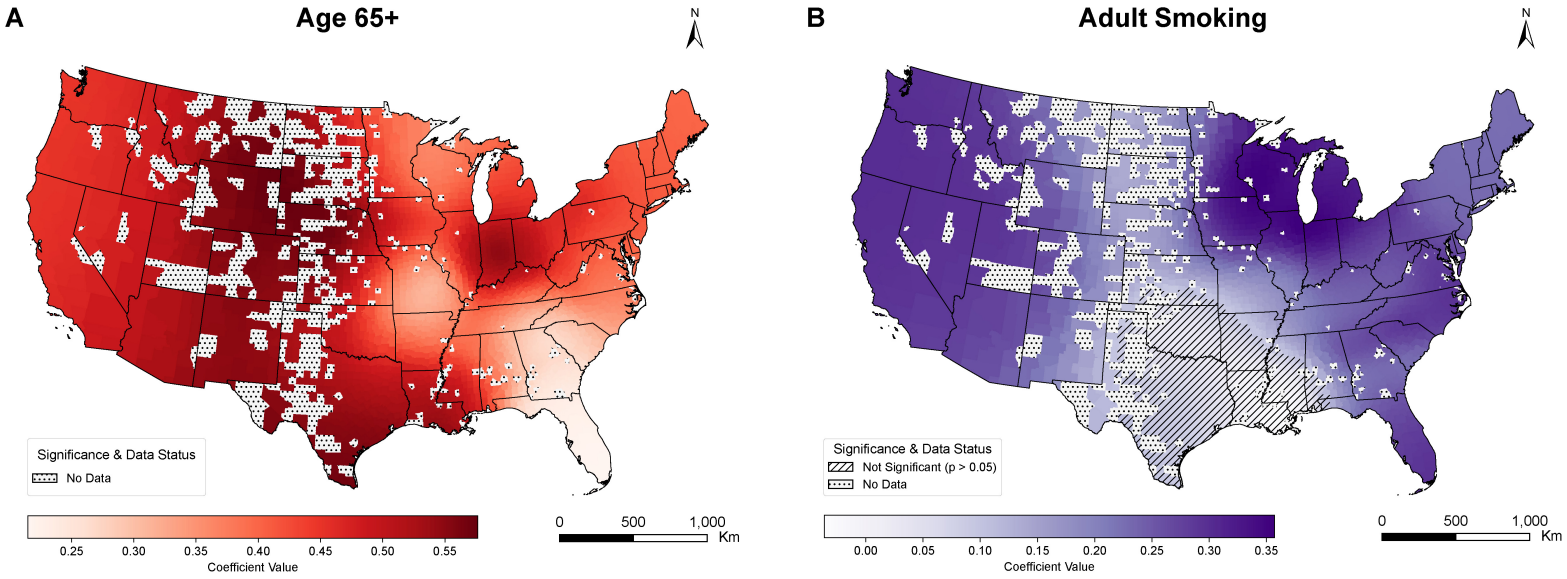
Spatial heterogeneity of the associations between key risk factors and RF-CLRD mortality rates derived from the MGWR model. The maps illustrate the spatial distribution of local regression coefficients for **(A)** the percentage of the population aged 65 years and older and **(B)** adult smoking prevalence. The color gradient represents the magnitude of the standardized coefficients (β), with darker colors indicating stronger positive associations. To distinguish statistical significance and data availability, hatched areas (slashes) indicate regions where the local association is statistically non-significant (*p* > 0.05), stippled areas (dots) indicate regions with missing data, and solid colored areas indicate regions with statistically significant associations (*p* < 0.05).

Figure 3 illustrates the distinct spatial non-stationarity of the two primary regional drivers, revealing that their impacts are geographically clustered rather than uniform. The population aged 65+ in Figure 3A exhibits a multi-centric high-impact pattern with three distinct hotspots including the Mountain West, the Midwest industrial belt, and the Western Gulf Coast. These intense clusters contrast with the Southeastern United States where the aging-mortality relationship remains significant but considerably weaker in magnitude. In comparison, adult smoking prevalence in Figure 3B displays a peripheral distribution with the strongest positive effects concentrated in the Upper Midwest, Pacific Northwest, and Southeast Atlantic Coast. This configuration surrounds a non-significant interior zone across the South Central United States where local coefficients suggest no statistical relationship between smoking and mortality variability.

## 4 Discussion

The application of MGWR in this analysis characterizes the spatial structure of the multifactor ecological niche for RF-CLRD mortality. The results indicate that risk factors operate at distinct spatial scales rather than functioning uniformly across the contiguous United States. By decomposing these spatial relationships, the model delineates a fundamental ecological structure where bioclimatic factors, air pollution, and social vulnerability act as broadly stable macro-regional stressors that define the baseline risk envelope, while demographic and behavioral factors function as localized amplifiers that generate specific high-mortality clusters.

These divergent bandwidths reflect the inherent spatial scales of the underlying physical and social processes. Bioclimatic extremes and PM_2.5_ operate globally due to macro-scale atmospheric dynamics and long-range transport that create contiguous environmental gradients (20). Similarly, the global scale of SVI reflects cross-regional macroeconomic structures (e.g., systemic poverty or industrial decline) that transcend county borders (21). Conversely, demographic aging and smoking operate regionally as they are driven by highly localized micro-processes. Aging clusters are shaped by specific county-level migration patterns, while smoking behaviors are strongly regulated by local policy environments (e.g., tobacco taxes) and community norms (22).

The identification of bioclimatic variables and PM_2.5_ as macro-regional drivers defines a consistent environmental envelope for respiratory failure. The global stability of coefficients for the maximum temperature of the warmest month and precipitation of the warmest quarter suggests that the physiological stress of hot and humid environments is a universal risk factor. Notably, the negative coefficient for mean diurnal range in the MGWR model indicates that lower temperature variation exacerbates the respiratory burden, likely because the absence of nocturnal cooling prevents physiological recovery in compromised patients (23). This climatic setup, combined with the positive association of PM_2.5_, aligns with evidence that extreme heat and particulate matter act as synergistic stressors rather than independent risks (24). Despite a certain degree of statistical correlation among retained climatic variables, they represent distinct biological pathways that cannot be mutually substituted. The co-occurrence of high humidity and heat facilitates the growth of biological allergens (25) while simultaneously inhibiting evaporative cooling, thereby intensifying the systemic airway inflammation induced by fine particulates (26, 27). Conversely, the retention of precipitation of the coldest quarter captures the contrasting physiological burden of cold and damp environments, which are well-documented triggers for reflex bronchoconstriction, airway mucosal cooling, and prolonged survival of respiratory pathogens (28). By disentangling these distinct mechanisms, the model demonstrates how this multifactorial stress creates a lethal environmental niche where the metabolic demand of thermoregulation exceeds the diminished respiratory capacity of RF-CLRD patients.

A critical finding from the MGWR analysis is the reclassification of the SVI as a macro-structural determinant. While the OLS model yielded a non-significant negative coefficient for SVI, the MGWR results reveal it as a significant global positive predictor with a bandwidth covering nearly all counties. This sign reversal is a classic resolution of “spatial confounding” rather than an artifact of instability (29). In OLS, unmeasured local determinants (e.g., municipal healthcare access) are forced into the global error term, creating omitted variable bias that distorts the true SVI effect. Conversely, MGWR captures these unobserved micro-level confounders through its highly localized intercept (bandwidth = 24), filtering out spatial noise to effectively unmask the true positive main effect of SVI (30). This suggests that socioeconomic deprivation functions as a pervasive structural component of the mortality niche rather than a local confounding factor (31). In regions characterized by high SVI, the lack of adaptive infrastructure such as air conditioning or high-quality housing envelopes prevents populations from decoupling their indoor environments from the outdoor climatic stress (32). Consequently, social vulnerability acts as a macro-scale force multiplier that lowers the physiological threshold at which the heat-pollution synergism leads to respiratory failure (33). This implies that structural inequality creates a broad susceptibility that renders large populations sensitive to climatic fluctuations regardless of local behavioral differences.

Within this broad macro-environmental and social envelope, demographic and behavioral determinants operate as distinct regional amplifiers. The MGWR analysis reveals that the population aged 65+ and adult smoking prevalence operate at significantly smaller spatial scales, with bandwidths of 475 and 414, respectively. The spatial non-stationarity of the smoking coefficient, ranging from −0.04 to 0.35, highlights that the impact of smoking is contingent upon the underlying environmental context. The observation of a hollow center in the South Central United States, where smoking shows no significant relationship with mortality, prompts us to hypothesize a potential saturation effect (34). We explicitly define this saturation as a threshold state where cumulative macro-environmental and structural stressors drive the baseline mortality risk to such an extreme that the additive impact of localized behavioral factors becomes statistically attenuated or “masked” (35). In these specific regions, the overwhelming burden of the climatic and social niche defined by heat, humidity, and structural deprivation may dominate the mortality risk, thereby diminishing the marginal explanatory power of smoking (36). However, interpreting this local non-significance through the lens of the model's own macro-environmental outputs warrants methodological caution to avoid circular reasoning. Consequently, this saturation effect should be viewed as a geographically generated hypothesis rather than a confirmed mechanism. This hypothesis also explains why smoking exhibits spatial saturation while PM_2.5_ and demographic aging do not. Bioclimatic extremes and PM_2.5_ function as pervasive foundational stressors that construct this high-risk baseline, often exhibiting non-linear saturation effects at extreme exposures (37). Meanwhile, aging represents an irreversible decline in intrinsic physiological resilience that universally elevates mortality. Neither of these foundational or intrinsic factors saturates in their geographical footprint. Conversely, because smoking operates as an individual-level behavioral amplifier, its relative impact is easily obscured in regions where the environmental baseline is overwhelmingly oppressive. In contrast, in regions where the environmental baseline is less oppressive, behavioral factors like smoking re-emerge as the primary discriminators of spatial mortality inequality (38).

The superiority of the MGWR framework is evidenced by the substantial improvement in model fit, with the Adjusted R2 increasing from 0.39 in OLS to 0.66, and the effective elimination of residual spatial autocorrelation (Moran's *I* = −0.085). By allowing the intercept to vary locally (bandwidth = 24), the model captures unobserved micro-scale factors inherent to specific counties, such as local hospital capacity or specific occupational hazards, which global models conflate with error (39). Therefore, in addition to addressing the macro-environmental burdens, public health measures should be tailored to the specific circumstances of different regions to better address the unique health risk factors and social vulnerabilities present in each area (40).

### 4.1 Limitations

This study is subject to limitations inherent to ecological analyses. The use of aggregate county-level data introduces the potential for ecological fallacy, as associations observed at the population level may not necessarily infer individual-level causality. Although the model controlled for PM_2.5_, unmeasured localized stressors such as indoor biomass burning or occupational dusts in mining regions could confound the results in specific clusters (41). While this study employs a cross-sectional spatial design that aggregates mortality and environmental data over a decade (2010–2019) to establish a definitive pre-pandemic baseline, it is important to contextualize this choice. In spatial epidemiology, computing rates for small geographical units (e.g., counties) often leads to unstable risk estimates due to stochastic variance, a challenge known as the “small numbers problem” (11). To mitigate this, temporal aggregation was utilized to stabilize the dependent variable and extract the reliable spatial structures required for the MGWR model (12). Furthermore, because RF-CLRD is driven by long-term cumulative exposures, decadal averages of PM_2.5_ and bioclimatic variables effectively capture the persistent “climatic niche” rather than transient meteorological noise (13, 14). While we recognize that this methodological trade-off inherently masks interannual temporal dynamics and precludes dynamic time-lag analyses, it is a standard and necessary compromise to ensure the robustness of macro-spatial estimations. Furthermore, this study relies on death certificate data which may lead to an underestimation of the actual mortality rate compared to hospital-based registries due to the pervasive classification bias and underreporting phenomena inherent in death registration systems (42). Additionally, relying solely on ecological model outputs to explain localized phenomena—such as the hypothesized spatial “saturation effect”—carries inherent inferential risks. Future research integrating independent stratified longitudinal cohorts and indoor environmental monitoring would be required to rigorously test these geographically generated hypotheses and establish the causal pathways between the macro-environmental niche and local amplifiers of respiratory health (43).

## 5 Conclusion

This study demonstrates that the spatial heterogeneity of RF-CLRD mortality across the contiguous United States is driven by a distinct dual-scale mechanism involving a regionally stable macro-environmental niche and localized demographic and health behavioral amplifiers. By utilizing the MGWR framework, we identified that the synergistic burden of bioclimatic factors, fine particulate matter, and social vulnerability operates as a broad and consistent stressor whereas aging populations and smoking prevalence function as spatially variable drivers. The superior performance of the MGWR model highlights the critical limitation of global models in capturing these scale-dependent relationships. Furthermore, these findings offer a critical epidemiological reference for international comparative studies and are especially applicable to nations undergoing rapid demographic transitions like China. By analyzing the exposure-response pathways in a post-industrial setting policymakers can better anticipate how the convergence of population aging and climate change will reshape the respiratory disease burden. Consequently effective public health interventions must transition from uniform national strategies to place-based precision policies. This entails a strategic divergence in resource allocation that prioritizes infrastructure-based heat adaptation and pollution mitigation in environmentally vulnerable regions while targeting community-based smoking cessation and geriatric care in specific high-risk clusters.

## Data Availability

Publicly available datasets were analyzed in this study. This data can be found here: the mortality data supporting the findings of this study are publicly available from the CDC WONDER database (https://wonder.cdc.gov/). Climatological data are available from the PRISM Climate Group (https://prism.oregonstate.edu/). Socioeconomic and demographic data can be accessed via the U.S. Census Bureau and the Agency for Toxic Substances and Disease Registry (ATSDR) (https://www.atsdr.cdc.gov/place-health/php/svi/svi-data-documentation-download.html). High-resolution PM2.5 data (V5.GL.02) were obtained from the Atmospheric Composition Analysis Group at Washington University in St. Louis (https://wustl.box.com/v/ACAG-V5GL0502-GWRPM25). The compiled dataset used for the MGWR analysis is available from the corresponding author upon reasonable request.
