# Supplementary material for: Mapping the mortality niche of chronic lower respiratory diseases complicated by respiratory failure: a multiscale spatial analysis of environmental and social drivers
Source: Front Public Health. 2026 May 26;14:1819428. doi: 10.3389/fpubh.2026.1819428 (PMC13246604; doi:10.3389/fpubh.2026.1819428)
Supplement: Supplementary file 1 [file Supplementary_File_1.docx]

**Figure S1. Distributional assessment and normalization of county-level RF-CLRD crude mortality rates**

The composite figure evaluates the statistical properties of the dependent variable before and after data transformation. The upper row displays the original crude mortality rate distribution characterized by a pronounced right skew in the histogram and significant deviation from the normal line in the Q-Q plot. The lower row illustrates the data following natural logarithmic transformation where the histogram exhibits a symmetrical bell curve and the Q-Q plot shows linear alignment with the theoretical quantiles confirming that the transformed variable satisfies the normality assumption required for subsequent regression analyses.


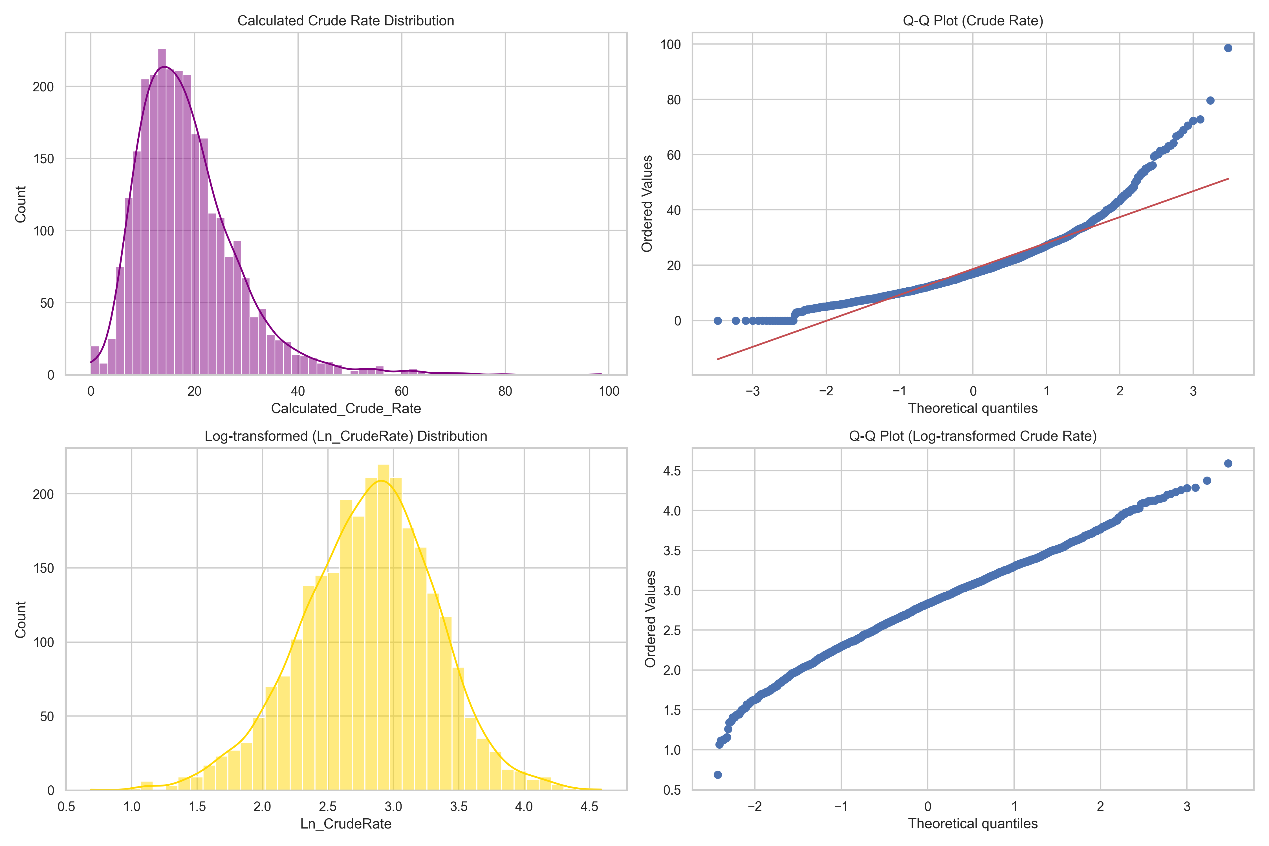


**Figure S2. Pairwise correlation matrix of candidate environmental and socioeconomic predictors**

The heatmap visualizes the Spearman rank correlation coefficients among all initial independent variables including bioclimatic factors air pollution and social determinants. The color scale ranges from deep blue representing strong negative correlations to dark red representing strong positive correlations while numerical values within each cell quantify the magnitude of the relationship.


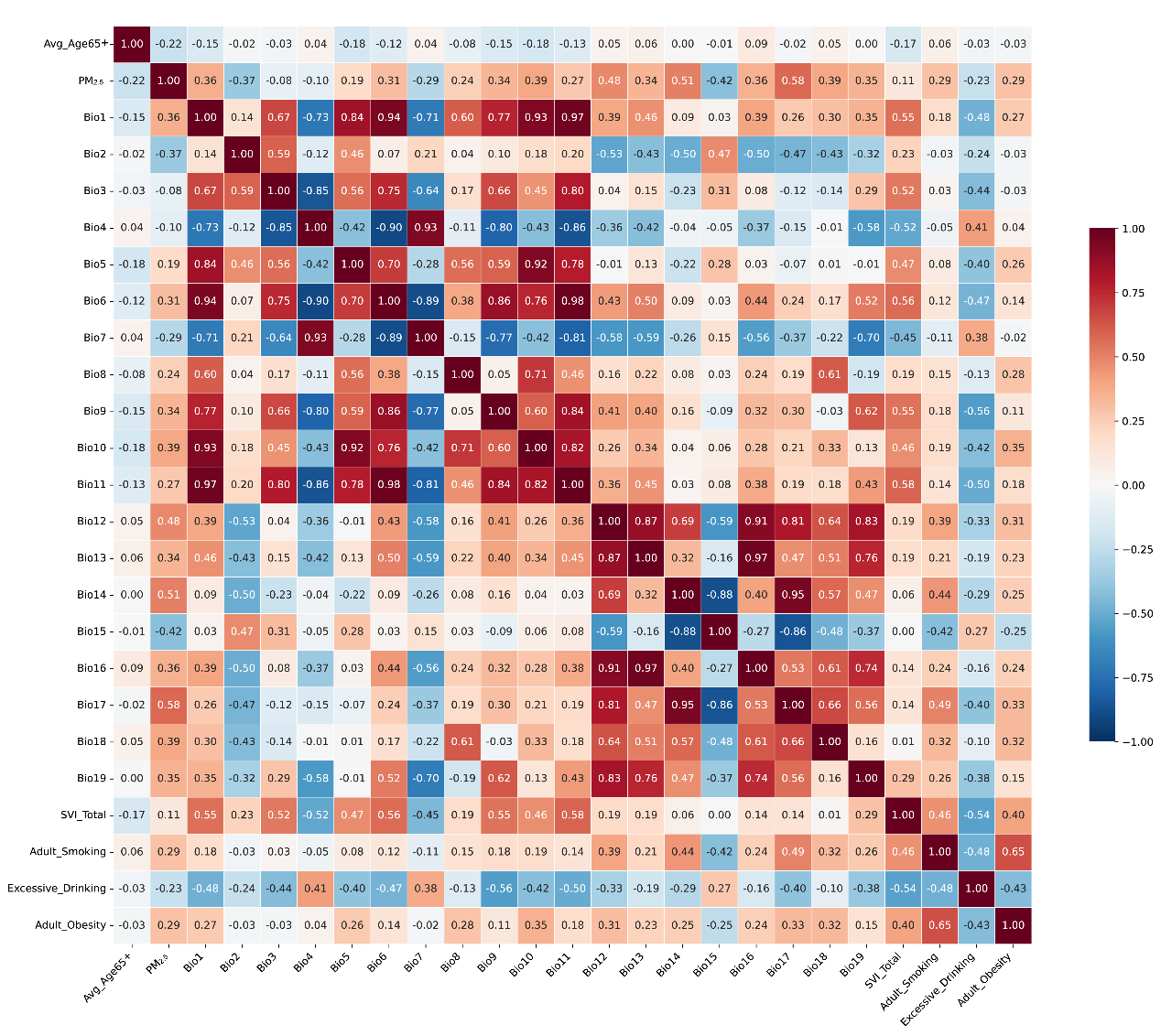


**Table S1. Descriptions of the 19 bioclimatic variables (BioClim).**

| **Code** | **Variable Name** | **Description** |
| --- | --- | --- |
| Bio1 | Annual mean temperature | The average annual temperature. |
| Bio2 | Mean Diurnal Range | The mean of the monthly temperature ranges (Max Temp - Min Temp). |
| Bio3 | Isothermality | The ratio of the mean diurnal range to the temperature annual range (BIO2 / BIO7) × 100. |
| Bio4 | Temperature Seasonality | The standard deviation of the monthly temperature estimates × 100. |
| Bio5 | Max Temperature of Warmest Month | The maximum temperature of the warmest month. |
| Bio6 | Min Temperature of Coldest Month | The minimum temperature of the coldest month. |
| Bio7 | Temperature Annual Range | The difference between the maximum temperature of the warmest month and the minimum temperature of the coldest month (BIO5 - BIO6). |
| Bio8 | Mean Temperature of Wettest Quarter | The mean temperature of the three wettest months. |
| Bio9 | Mean Temperature of Driest Quarter | The mean temperature of the three driest months. |
| Bio10 | Mean Temperature of Warmest Quarter | The mean temperature of the three warmest months. |
| Bio11 | Mean Temperature of Coldest Quarter | The mean temperature of the three coldest months. |
| Bio12 | Annual Precipitation | The total annual precipitation. |
| Bio13 | Precipitation of Wettest Month | The precipitation of the wettest month. |
| Bio14 | Precipitation of Driest Month | The precipitation of the driest month. |
| Bio15 | Precipitation Seasonality | The coefficient of variation of the monthly precipitation estimates. |
| Bio16 | Precipitation of Wettest Quarter | The total precipitation of the three wettest months. |
| Bio17 | Precipitation of Driest Quarter | The total precipitation of the three driest months. |
| Bio18 | Precipitation of Warmest Quarter | The total precipitation of the three warmest months. |
| Bio19 | Precipitation of Coldest Quarter | The total precipitation of the three coldest months. |

**Table S2.** **Stepwise exclusion process and diagnostics for candidate independent variables.**

The table details the rigorous stepwise exclusion process for all initial candidate variables prior to regression modeling. Variables were first evaluated based on their absolute Spearman correlation with RF-CLRD mortality (|r|). Highly collinear pairs (|r| > 0.75) were resolved by excluding the predictor with the weaker association. Subsequent variance inflation factor (VIF) screening was applied to the remaining covariates. Dashes (—) indicate that the variable successfully passed the specific screening phase. Ultimately, thirteen covariates were retained in the final regression model, with all exhibiting VIF values significantly below the conservative threshold of 5.

| **Variable** | **Bivariate Correlation with Mortality (\|r\|)** | **Exclusion Rationale** | **VIF (Post-Spearman Screening)** | **Selection Outcome** |
| --- | --- | --- | --- | --- |
| Avg_Age65+ | 0.471 | — | 1.20 | Retained |
| PM2.5 | 0.022 | — | 1.96 | Retained |
| Bio1 | 0.063 | r > 0.75 with Bio7 |  | Excluded |
| Bio2 | 0.144 | — | 2.74 | Retained |
| Bio3 | 0.034 | — | 16.65 | Excluded |
| Bio4 | 0.049 | r > 0.75 with Bio7 |  | Excluded |
| Bio5 | 0.029 | — | 3.81 | Retained |
| Bio6 | 0.075 | r > 0.75 with Bio7 |  | Excluded |
| Bio7 | 0.082 | — | 3.53 | Retained |
| Bio8 | 0.074 | — | 4.34 | Retained |
| Bio9 | 0.009 | r > 0.75 with Bio7 |  | Excluded |
| Bio10 | 0.056 | r > 0.75 with Bio8 |  | Excluded |
| Bio11 | 0.059 | r > 0.75 with Bio7 |  | Excluded |
| Bio12 | 0.051 | r > 0.75 with Bio19 |  | Excluded |
| Bio13 | 0.009 | r > 0.75 with Bio19 |  | Excluded |
| Bio14 | 0.072 | — | 2.93 | Retained |
| Bio15 | 0.047 | r > 0.75 with Bio14 |  | Excluded |
| Bio16 | 0.020 | r > 0.75 with Bio19 |  | Excluded |
| Bio17 | 0.064 | r > 0.75 with Bio14 |  | Excluded |
| Bio18 | 0.022 | — | 3.85 | Retained |
| Bio19 | 0.064 | — | 3.43 | Retained |
| SVI_Total | 0.077 | — | 2.41 | Retained |
| Adult_Smoking | 0.366 | — | 2.55 | Retained |
| Excessive_Drinking | 0.218 | — | 2.35 | Retained |
| Adult_Obesity | 0.222 | — | 2.31 | Retained |
